# Differences in Trocar Positioning within the Vertebral Body Using Two Different Positioning Methods: Effect on Trainee Performance

**DOI:** 10.1155/2011/830961

**Published:** 2011-12-08

**Authors:** Atsushi Komemushi, Kenji Takizawa, Norimitsu Tanaka, Misako Yoshimatsu, Kunihiro Yagihashi, Yukihisa Ogawa, Atsuko Fujikawa, Iwao Uejima, Yuya Koike, Taiji Tamura, Makoto Takahashi, Jun Koizumi, Koichiro Yamakado, Seishi Nakatsuka, Tetsuya Yoshioka, Shozo Hirota, Kenji Nakamura, Yasuo Nakajima, Sachio Kuribayashi, Shuji Kariya, Noboru Tanigawa, Satoshi Sawada

**Affiliations:** ^1^Department of Radiology, Kansai Medical University, Moriguchi, Osaka 570-8507, Japan; ^2^Department of Radiology, St. Marianna University School of Medicine, Japan; ^3^Department of Radiology, Kurume University School of Medicine, Japan; ^4^Department of Radiology, Self-Defense Forces Central Hospital, Japan; ^5^Terumo Medical Pranex, R & D Center, Terumo Corporation, Japan; ^6^Department of Radiology, Tokai University School of Medicine, Japan; ^7^Department of Radiology, Mie University School of Medicine, Japan; ^8^Department of Diagnostic Radiology, Keio University School of Medicine, Japan; ^9^Department of Radiology, Narumi Hospital, Japan; ^10^Department of Radiology, Hyogo College of Medicine, Japan; ^11^Department of Radiology, Daito Central Hospital, Japan

## Abstract

*Purpose*. To evaluate the educational effect of the Japanese Society of Interventional Radiology 7th Academic Summer Seminar from a technical perspective. *Materials and Methods*. Nineteen trainees participated in the seminar. The seminar consisted of vertebroplasty trainings using swine with the single-plane landmark method and with the ISOcenter Puncture (ISOP) method. All trainees were advised by an instructor as they operated the instruments and punctured the vertebra. For each trainee, the accuracy in the final position of the needle tip of the initial puncture in each swine training was evaluated. *Results*. Error in the final position of the needle tip of ≥5 mm from the target puncture site occurred in the lateral direction in 42% (8/19) of trainees with the landmark method and 5% (1/19) with the ISOP method. No error ≥5 mm occurred in the vertical or anteroposterior directions. In terms of puncture accuracy, error in the lateral direction was significantly lower with the ISOP method than with the landmark method (2.2 ± 1.5 mm versus 5.6 ± 3.2 mm). *Conclusion*. This seminar was effective training for trocar placement for beginners. The puncture was more accurate with the ISOP method than with the landmark method.

## 1. Introduction

Percutaneous vertebroplasty (PVP) is spreading rapidly and the need for training of doctors new to the procedure is increasing. However, training beginners in an invasive procedure in clinical settings is difficult from the perspective of patient safety. Hands-on seminars using cadavers [1] are conducted as PVP training for beginners, but in Japan the use of cadavers in training for interventional radiologic procedures is difficult because of legal restrictions. As training for beginners, the Japanese Society of Interventional Radiology held a hands-on seminar for PVP under fluoroscopic guidance at its 7th Academic Summer Seminar. This seminar consisted of lectures and practical training using swine.

The purpose of this study was to evaluate the educational effect of this seminar from a technical perspective. 

## 2. Materials and Methods

This study was approved by an ethics committee. The trainees were not informed that the accuracy of punctures would be evaluated and reported in a journal article until the end of the seminar. Consent was obtained from all trainees.

### 2.1. The Japanese Society of Interventional Radiology 7th Academic Summer Seminar

The Seminar was held by the Japanese Society of Interventional Radiology at Terumo Medical Pranex (Kanagawa, Japan) on July 25-26, 2009.

Nineteen male trainees (age: mean 39.2 years, median 37 years, range 27–51 years; years since acquiring medical license: mean 12.2, median 10, range 3–26) participated in the seminar. Eight of the 19 (42%) were Board Certified Interventional Radiologists by the Japanese Society of Interventional Radiology, and 8/19 (42%) had experience with PVP.

The seminar consisted of a total of 5 hours and 20 minutes of lectures on PVP and three types of practical training, each lasting 120 minutes. The content of the lectures was as follows: What is PVP? 30 minutes; puncture-landmark method and ISOcenter Puncture method (ISOP) method: 30 minutes; Experimental animal anatomy: 15 minutes; examination prior to PVP—interview, pathological findings, diagnostic imaging: 20 minutes; PVP results and complications: 20 minutes; complications, especially new fractures in adjacent vertebrae: 20 minutes; case conference: 60 minutes; QOL evaluation: 40 minutes; clinical considerations of osteoporosis and vertebral fracture: 30 minutes; frequently asked questions: 20 minutes; recent topics in PVP: 20 minutes; general review: 15 minutes. All trainees learned detailed knowledge about PVP including vertebral bony landmarks, transpedicular approach, para-pedicular approach unilateral approach, bilateral approach, and technique of adjust needle placement depending upon the appearance of the vertebral body (degree of compression, wedging, angulation of the endplates, etc.).

The following three types of practical training were conducted for 120 minutes each: PVP training with the single-plane landmark method using swine, PVP training with the ISOP method using swine, and a simulated procedure using a human mannequin. The trainees were divided into three groups and performed the three types of practical training in turn. All trainees were advised by an instructor as they operated the instruments and punctured the vertebra. In the vertebroplasty training using swine, the puncture target point was set at the anterior third of the vertebra in the anteroposterior direction and the center of the vertebra vertically and laterally.

### 2.2. Practical Training

Six female pigs weighing 51.0–53.9 kg (mean weight, 52.4 kg ± 2.2) were studied. All animals were carefully maintained and cared for before and during the experiment in accordance with the National Institutes of Health Guidelines for the Use of Laboratory Animals.

All procedures were performed under general anesthesia. Animals were placed prone, and then general anesthesia was induced with an intramuscular injection of ketamine hydrochloride (12.5 mg/kg, Ketalar Intramuscular 500 mg; Daiichi Sankyo Co. Ltd., Tokyo, Japan) and maintained with sevoflurane (5%, Sevofrane; Maruishi Pharmaceutical Co. Ltd., Osaka, Japan) administered using a mask. After anesthetic administration, an endotracheal tube was inserted and anesthesia was maintained with sevoflurane (5%), nitrous oxide (3 L/min), and oxygen (3 L/min).

#### 2.2.1. Vertebroplasty Training with the Single-Plane Landmark Method [2, 3] Using Swine: 120 Minutes

NT was an instructor of this training. An anteroposterior image of the vertebra was viewed using a single-plane fluoroscopy unit (Allura Xper FD20, Philips Healthcare Japan, Tokyo, Japan). The viewing field was adjusted on the cephalocaudal axis so that the vertebral arch was projected in the center of the vertebral body. The viewing field was adjusted laterally so that the pedicle of the vertebral arch was viewed from the front.

The following procedure was followed for PVP with the single-plane landmark method. Using the transpedicular route, the needle is centered at the 10 o'clock position over the left pedicle and at 2 o'clock over the right pedicle with the help of a long needle holder, thus avoiding radiation to the operator's hands. The needle should also be medialized through the cylinder of the pedicle to reach the middle of the vertebra. Once a clear image is obtained of the needle in the pedicle, and the position is considered ideal on the front view, the needle is advanced under the guidance of lateral fluoroscopy. The tip of the needle should lie beyond the midpoint of the vertebral body on the lateral view. The ideal needle-tip position is in the anterior third of the vertebra in the anteroposterior direction and in the center vertically and laterally.

#### 2.2.2. Vertebroplasty Training with the ISOP Method [4, 5] Using Swine: 120 Minutes (Figure 1)

 KT was an instructor of this training. In this method, the isocenter of the C-arm radiographic system is the center of the radiation field and the center of the C-arm rotation. Thus, the isocenter always remains at the center of the radiation field as well as the center of the monitor screen, no matter how the C-arm is rotated. The ISOP method applies this principle, and therefore, adjusting the puncture target to the position of the isocenter becomes essential.

A square of 20 × 20 pixels showing the isocenter was displayed in the center of the monitor of the single-plane fluoroscopy unity (Infinex Celeve CC, Toshiba Medical Inc., Tokyo, Japan) as the isocenter marker.

PVP procedures with the ISOP method consist of the following three processes.

**(a) Positioning**

The examining table is moved with the monitor screen used for fluoroscopic viewing of the front and lateral images until the puncture target overlaps the isocenter marker. In the training, the puncture target was taken to be in the anterior third of the vertebra in the anteroposterior direction and at the center vertically and laterally.

**(b) Rotation of the C-Arm**

The C-arm is rotated in a three-dimensional direction until the isocenter marker overlaps the center of the pediculus arcus vertebral image.

**(c) Puncture**

Puncture is conducted under fluoroscopic guidance, with the isocenter marker as the target. Following penetration of the bony cortex of the vertebral arch pedicle, the needle is advanced up to the target indicated by the isocenter marker, under frontal and lateral fluoroscopic viewing.

### 2.3. Evaluation

**Figure 2:**
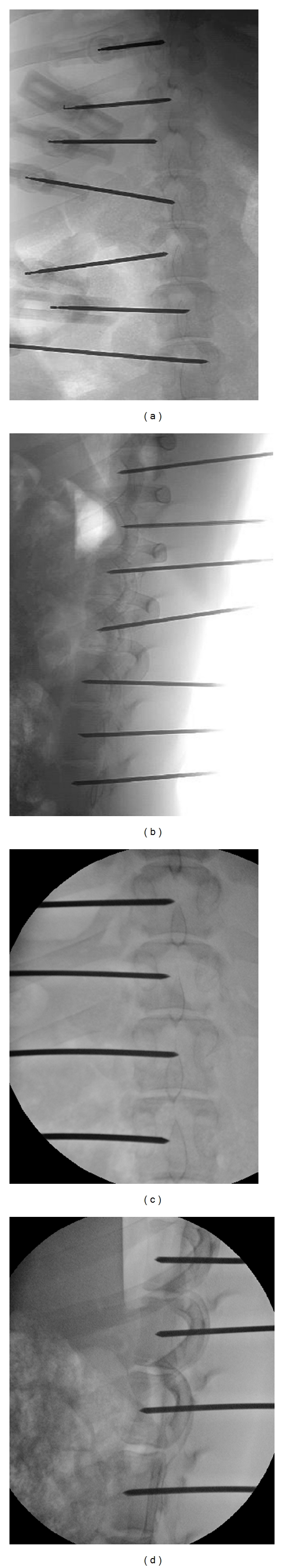
Plain radiographs for evaluation. (a) Landmark method, frontal view. (b) Landmark method, lateral view. (c) ISOP method, frontal view. (d) ISOP method, lateral view.

For each trainee, the accuracy in the final position of the needle tip of the initial puncture in each swine training was evaluated on plain radiographs. The radiograph was calibrated with the thickness of the puncture needle as the reference. Lateral puncture error was measured using an anteroposterior view plain radiograph, and vertical and anteroposterior puncture errors were measured using a lateral view plain radiograph (Figure 2). Statistical evaluation was performed using a two-tailed unpaired Student's *t*-test, with *P* < 0.05 taken to indicate statistical significance.

## 3. Results

Trainees had no notable difficulties during the seminar. All 19 trainees completed the seminar successfully.

Error in the final position of the needle tip of ≥5 mm from the target puncture site occurred in the lateral direction in 42% (8/19) of trainees with the landmark method and 5% (1/19) with the ISOP method (Table 1). No error ≥ 5 mm occurred in the vertical or anteroposterior directions. The maximum puncture error in the final position of the needle tip with the traditional method and ISOP method was 13.1 mm and 5.5 mm, respectively, in the lateral direction, 4.5 mm and 2.8 mm in the vertical direction, and 4.3 mm and 2.6 mm in the anteroposterior direction. In terms of puncture accuracy, error in the lateral direction was significantly lower with the ISOP method than with the landmark method (2.2 ± 1.5 mm versus 5.6 ± 3.2 mm). No significant difference was seen in vertical error (1.1 ± 0.8 mm versus 1.6 ± 1.4 mm) or anteroposterior error (1.1 ± 0.9 mm versus 1.7 ± 1.1 mm) (Table 2).

## 4. Discussion

Performing PVP in clinical settings requires both knowledge of the procedure and the skills to carry it out. At the Japanese Society of Interventional Radiology 7th Academic Summer Seminar, the trainees obtained the knowledge needed to perform PVP through lectures totaling 5 hours and 20 minutes, and they were taught the necessary skills to perform PVP during a total of 6 hours of practical training.

In one type of training procedure, PVP was simulated using a human mannequin, and in the other two types, trainees were guided in performing PVP with two types of puncture technique using swine. In the single-plane landmark method, the puncture needle is guided inside the vertebral body with reference to anatomical structures, with the pedicle of the vertebral body viewed fluoroscopically from the front [2, 3]. The ISOP method is a puncture method developed by Takizawa et al. [4, 5]. By locating the puncture target in the isocenter, accuracy is high even using a single-plane fluoroscopy unit, and the procedure can be performed easily. In this study, we evaluated the puncture accuracy in the final position of the needle tip of students' initial puncture in practical training for PVP using swine.

The largest puncture errors occurred with the landmark method in the lateral, vertical, and anteroposterior directions. However, the maximum error in the lateral direction was 13.1 mm, which is within the range of acceptable error for beginners' first punctures. The landmark method is a puncture method that aims to puncture the vertebral body with reference to anatomical structures on a path that does not damage the spinal canal. The difference in shape of the vertebrae between humans and swine is thought to have been difficult to accurately guide the puncture needle in the lateral direction.

The ISOP method is a puncture assistance method that aims to facilitate easy and accurate puncture by fixing the puncture target on the isocenter. The ISOP method is broadly applicable to all types of C-arms by only putting small marker on the center of monitor screen without special hardware and software. In punctures using the ISOP method, the error, even for the first punctures of beginners, was very small, at 2.2 mm in the lateral direction and 1.1 mm in the vertical and anteroposterior directions. The ISOP method therefore appears useful as a puncture method for beginner operators.

 There were some limitations in this study. First, there was not a separate analysis of the trainees with and without previous PVP experience. Second, it was anatomically different in vertebrae between patients and swine. Swine had smaller pedicles and vertebrae than patients.

All trainees at the Japanese Society of Interventional Radiology 7th Academic Summer Seminar successfully completed the training without difficulties. All were able to puncture the vertebral body with acceptable accuracy in their first puncture under appropriate guidance with either the landmark or ISOP method. This seminar was an effective training for trocar placement for beginners. The puncture was more accurate with the ISOP method than with the landmark method.

## Figures and Tables

**Figure 1:**
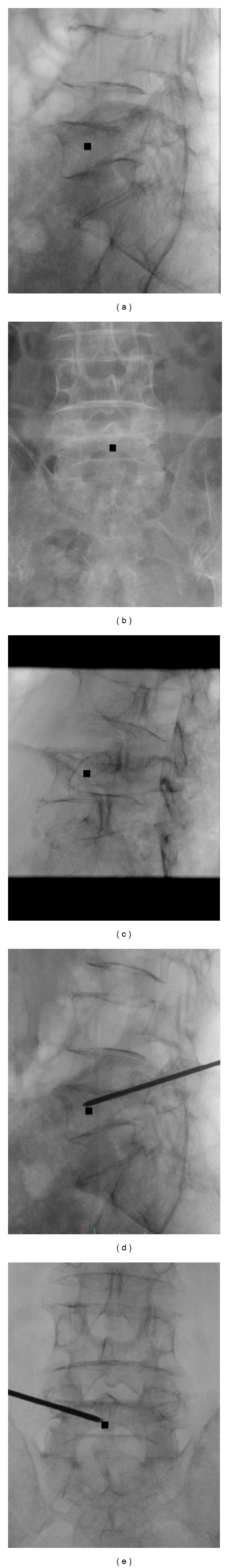
ISOcenter puncture method. (a) Positioning: lateral view. (b) Positioning: frontal view. The examining table is moved with the monitor screen used for fluoroscopic viewing of the front and lateral images until the puncture target overlaps the isocenter marker. (Arrow: Isocenter marker.) (c) Rotation of the C-arm. The C-arm is rotated in a three-dimensional manner until the isocenter marker overlaps the center of the pediculus arcus vertebral image. (d) Puncture: lateral view. (e) Puncture: frontal view. Puncture is conducted while targeting the isocenter marker under fluoroscopic guidance. Following penetration of the bony cortex of the vertebral arch pedicle, the needle is advanced up to the target indicated by the isocenter marker, under frontal and lateral fluoroscopic viewing.

**Table 1:** Frequency of error of ≥5 mm from the puncture target.

	Lateral	Vertical	Anteroposterior
Landmark method (*n* = 19)	42% (8/19)	0% (0/19)	0% (0/19)
ISOP method (*n* = 19)	5% (1/19)	0% (0/19)	0% (0/19)

**Table 2:** Mean ± standard deviation and maximum error from the puncture target.

	Lateral	Vertical	Anteroposterior
Landmark method (*n* = 19)	5.6 ± 3.2 (max: 13.1 mm)	1.6 ± 1.4 (max: 4.5 mm)	1.7 ± 1.1 (max: 4.3 mm)
ISOP method (*n* = 19)	2.2 ± 1.5 (max: 5.5 mm)	1.1 ± 0.8 (max: 2.8 mm)	1.1 ± 0.9 (max: 2.6 mm)
Student's *t*-test	*P* < 0.001	N.S.	N.S.
